# Research on the Effect of Oriental Fruit Moth Feeding on the Quality Degradation of Chestnut Rose Juice Based on Metabolomics

**DOI:** 10.3390/molecules28207170

**Published:** 2023-10-19

**Authors:** Tingyuan Ren, Bei Li, Fangyan Xu, Zhen Chen, Mintao Lu, Shuming Tan

**Affiliations:** School of Liquor and Food Engineering, Guizhou University, Guiyang 550025, China; libei20220917@163.com (B.L.); xufy980502@163.com (F.X.); cz090511@163.com (Z.C.); lumintao0606@163.com (M.L.); smtan@gzu.edu.cn (S.T.)

**Keywords:** oriental fruit moth, chestnut rose juice, bitterness, functional ingredients, metabolomics

## Abstract

As a native fruit of China, chestnut rose (*Rosa roxburghii* Tratt) juice is rich in bioactive ingredients. Oriental fruit moth (OFM), *Grapholita molesta* (Busck), attacks the fruits and shoots of *Rosaceae* plants, and its feeding affects the quality and yield of chestnut rose. To investigate the effects of OFM feeding on the quality of chestnut rose juice, the bioactive compounds in chestnut rose juice produced from fruits eaten by OFM were measured. The electronic tongue senses, amino acid profile, and untargeted metabolomics assessments were performed to explore changes in the flavour and metabolites. The results showed that OFM feeding reduced the levels of superoxide dismutase (SOD), tannin, vitamin C, flavonoid, and condensed tannin; increased those of polyphenols, soluble solids, total protein, bitterness, and amounts of bitter amino acids; and decreased the total amino acid and umami amino acid levels. Furthermore, untargeted metabolomics annotated a total of 426 differential metabolites (including 55 bitter metabolites), which were mainly enriched in 14 metabolic pathways, such as flavonoid biosynthesis, tryptophan metabolism, tyrosine metabolism, and diterpenoid biosynthesis. In conclusion, the quality of chestnut rose juice deteriorated under OFM feeding stress, the levels of bitter substances were significantly increased, and the bitter taste was subsequently enhanced.

## 1. Introduction

Chestnut rose (*Rosa roxburghii* Tratt) of the *Rosaceae* family is a rare and native fruit in China, and its juice is rich in functional active components, such as vitamin C, superoxide dismutase (SOD), and terpenoids. Chestnut rose is also known as the “King of Vitamin C” because of its high vitamin C content [1]. As the geographic origin of chestnut rose, Guizhou Province in China has been focusing on chestnut rose plantation, exploitation and utilization [2,3]. Studies have shown that chestnut rose juice has reproductive protection, antioxidant, anti-inflammatory, anticancer, and immunity enhancement properties [4,5,6]. With the rising social recognition of chestnut rose juice, a variety of chestnut rose products, including chestnut rose drinks, chestnut rose wines, and chestnut rose preserved fruits are attractive for the public. However, chestnut rose juice has a highly bitter taste, since it contains high levels of polyphenols and flavonoids. This has become an obstacle to the consumption of chestnut rose products. Although some enterprises have adopted techniques to remove the astringent and bitter flavours during the processing of chestnut rose products, there are some side effects, such as serious nutrient loss, complicated processing, and poor quality improvement [7]. Plant bitterness is derived from bitter substances. Increasing evidence has shown that in addition to inherent bitter substances, when subjected to external stress, the plant’s defence system activates the synthesis of additional bitter substances and enhances the activity of the original bitter substances to resist invasion from pests, diseases, mechanical injuries, and drought [8,9].

Oriental fruit moth (OFM), *Grapholita molesta* (Busck) (*Lepidoptera*: *Tortricidae*), is a common pest of the roots and fruits of *Rosaceae* plants [10]. As the main pest of chestnut rose, OFM feeding directly disturbs the quality and yield of chestnut rose [11,12]. Early feeding of OFM is one of the main causes of fruit shedding, and OFM feeding in the middle and late stages of maturity is problematic for chestnut rose processing [11]. Statistical data from Guizhou chestnut rose juice processing enterprises in recent years showed that 20~60% of chestnut rose suffers from OFM feeding stress. Because it is difficult to distinguish chestnut rose fruit undergoing OFM feeding from normal fruit on their appearance, equipment and technology specifically designed for screening fruit eaten by OFM is unavailable. Although OFM feeding did not significantly change the appearance of chestnut rose fruit, the effect on its quality was unclear. Our previous studies have revealed that compared with normal chestnut rose juice, there is significantly increased bitterness, astringency, and decreased storage stability in chestnut rose juice from fruits subjected to OFM feeding stress. However, the mechanism of quality deterioration of juice from chestnut rose eaten by OFM is unclear. Therefore, it is necessary to explore the basis of the nutrient and flavour deterioration in chestnut rose juice under OFM feeding stress. This will provide theoretical guidance for the planting, management, and processing of high-quality chestnut rose.

In this study, function, flavour deterioration, and metabolic pathways of chestnut rose juice deterioration under OFM feeding stress were explored by using physiological and biochemical methods to determine the functional active components of chestnut rose juice after OFM feeding. Sensory evaluations of flavour and amino acid detection were carried out by an electronic tongue and automatic amino acid analyser, and changes in metabolic pathways were revealed through nontargeted metabolomics.

## 2. Results and Discussion

### 2.1. Effects of OFM Feeding on Functional Active Ingredients in Chestnut Rose Juice

The normal fruit and those subjected to OFM feeding could not be distinguished based on their appearance. However, after cutting the fruit open, it was observed that the inside of the latter was “blackened” (Figure S1), and the worm fruit rate reached 27.68% in these fruits. As shown in Table 1, there was no significant difference in weight, the ratio of transverse to longitudinal diameters, or total colour, but the juice yield of normal fruit (67.19%) was significantly higher than that of fruit subjected to OFM feeding (55.36%). In the fruit juice from fruit subjected to OFM feeding, there was a decrease in SOD and tannin content, especially that of vitamin C, condensed tannins, and flavones; there were also increased levels of polyphenols, soluble solids, and total protein. These results indicate that OFM feeding seriously degraded the quality of chestnut rose juice.

### 2.2. Effects of OFM Feeding on the Flavour of Chestnut Rose Juice

**Figure 1:**
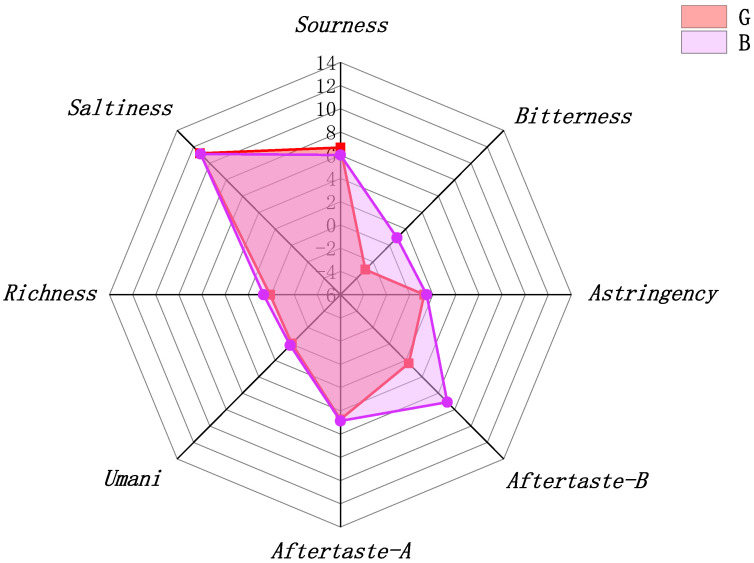
Radar chart of electronic tongue taste measurements of chestnut rose juice.

The sensor signals of samples G and B were collected using an electronic tongue, and the conversion values of eight taste indicators positively related to flavour intensity, including bitterness, astringency, aftertaste-B (bitter aftertaste), aftertaste-A (astringent aftertaste), umami, umami aftertaste, and saltiness, were obtained. The negative values indicate that the taste failed for the detected sensor. A drawing of the radar map from conversion values is shown in Figure 1, which visualizes the difference in taste between the two samples. OFM feeding had no significant effect on the saltiness and umami values of chestnut rose juice, but it decreased the acidity value, improved astringency and aftertaste-A, and significantly enhanced bitterness and aftertaste-B. These results suggest that chestnut rose produces bitter astringent substances to resist OFM feeding stress.

### 2.3. Effects of OFM Feeding on Amino Acid Profiles in Chestnut Rose Juice

**Figure 2:**
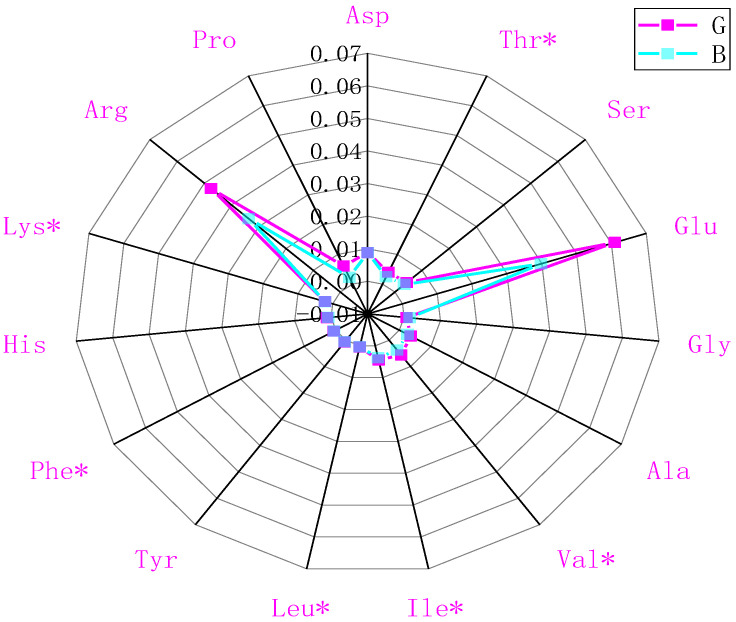
Comparison of the amino acid content of chestnut rose juice. ‘*’ represents essential amino acids.

The amino acid profiles of chestnut rose juice were measured using an automatic amino acid analyser (proline content was determined at a wavelength of 440 nm; other amino acid contents were detected at a wavelength of 570 nm). Fifteen amino acids were detected, six of which (threonine, valine, isoleucine, leucine, phenylalanine, lysine) were essential and accounted for 11.50% and 12.27% of the total amino acids in samples G and B, respectively. An amount of 12 amino acids, including threonine, serine, alanine, valine, isoleucine, leucine, tyrosine, phenylalanine, histidine, proline, and especially arginine and glutamic acid, were significantly decreased by OFM feeding, but the amounts of aspartate and lysine were almost unchanged (Figure 2). Therefore, the increased bitterness of the chestnut rose juice may be attributed to decreased umami amino acid content (such as arginine and glutamate), an increased proportion of bitter amino acids (such as histidine, isoleucine, tyrosine, phenylalanine, valine, leucine, etc.), And an increased proportion of bitter amino acids (such as histidine, isoleucine, tyrosine, phenylalanine, valine, leucine, etc.).

### 2.4. Effects of OFM Feeding on Metabolites in Chestnut Rose Juice

#### 2.4.1. Nontargeted Metabolomics Analysis

Furthermore, to investigate the changes in flavour and in the characteristic substances in chestnut rose juice from fruits subjected to OFM feeding stress, nontargeted metabolomics based on LC-QTOF were performed to analyse metabolite changes in sample G and sample B. A total of 3898 metabolites were identified, 2398 of which were detected in positive ion mode, and 1500 of which were detected in negative ion mode. In this study, FC ≥ 1 (upregulated), FC ≤ 0.5 (downregulated), VIP ≥ 1, and *p* < 0.05 were set as screening conditions. A total of 426 differential metabolites were identified, including 130 in positive ion mode and 296 in negative ion mode. The identified metabolites were mainly enriched in amino acid metabolism, biosynthesis of other secondary metabolites, and metabolism of terpenes and polyketones. These metabolites may represent the material basis of the chestnut rose juice quality reduction following OFM feeding.

#### 2.4.2. Principal Component Analysis (PCA)

**Figure 3:**
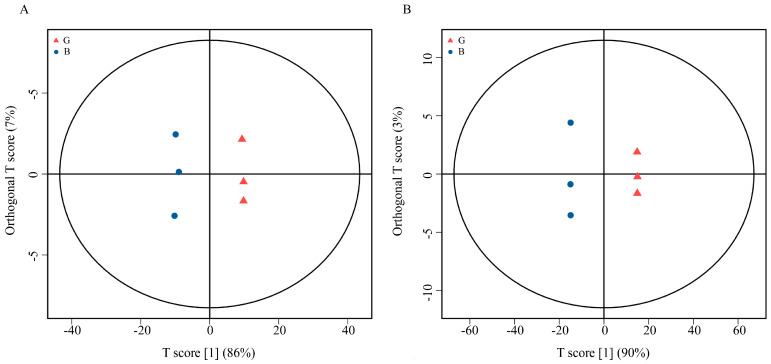
OPLS_DA analyse. The x_axis (t1) represents the prediction component (inter_group difference component), the y_axis (t2) represents the orthogonal component (intra_group difference component), and the transverse y_axis percentage represents the proportion of this component in the total variance. (**A**) Positive ion model; (**B**) Negative ion model.

Principal component analysis (PCA) of samples G and B was performed before the differential metabolite analysis. The results showed that there was a high degree of differentiation between the two samples in the positive and negative ion modes (Figure S2). OPLS-DA also showed that there was a large degree of separation between sample G and sample B in positive and negative ion modes. The prediction parameters of the evaluation model include R2X, R2Y, and Q2Y, where R2 represents the interpretability of the model variable, and Q2 represents the predictability of the model. (R2X = 0.934, R2Y = 0.998, Q2Y = 0.958 in positive ion mode, R2X = 0.958, R2Y = 1 in negative ion mode; Q2Y = 0.999), indicating a significant difference in metabolites in the two samples (Figure 3). The permutation test of the OPLS-DA model demonstrated that there was high independence between the training set and the test set, indicating that the OPLS-DA model was reliable (Figure S3).

#### 2.4.3. Cluster Analysis of Differential Metabolites

**Figure 4:**
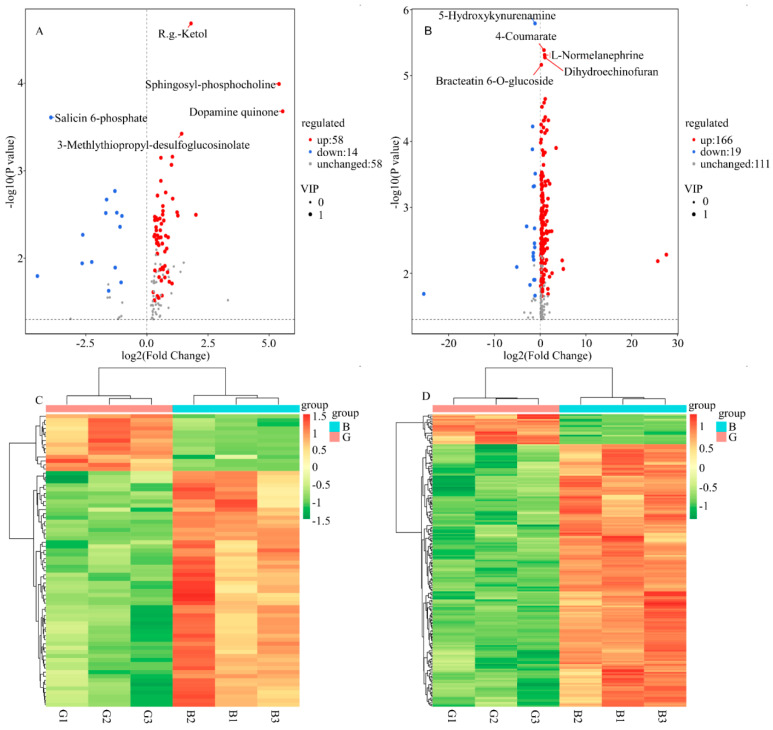
(**A**,**B**) Differentially accumulating metabolites between ‘G’ and ‘B’. A total of 426 metabolites were identified on the volcanic plot. (**C**,**D**) Cluster analysis of metabolites from samples of ‘G’ and ‘B’. The colour indicates the level of accumulation of each metabolite, from low (green) to high (orange). (**C**) Positive ion model; (**B**,**D**) Negative ion model.

The volcano plots showed that of the 130 different metabolites identified under the positive ion model, 72 metabolites had significant changes, including 58 upregulated metabolites (such as R.g.-ketol, sphingosyl-phosphocholine, dopamine quinone, and 3-Methylthiopropyl-desulfogluco-sinolate) and 14 downregulated metabolites (such as salicin 6-phosphate). Of the 296 different metabolites identified under the negative ion model, 185 metabolites had significant changes, including 166 upregulated metabolites (such as 4-coumarate, L-normelanephrine, dihydroechinofuran, and bracteatin 6-O-glucoside) and 19 downregulated metabolites (such as 5-hydroxykynurenine) (Figure 4A,B). The clustering heatmaps of differential metabolites showed that metabolites were similar within the groups, but there were differences between the groups (Figure 4C,D), suggesting that sample G and sample B have different metabolic characteristics.

#### 2.4.4. Enrichment Analysis of Differential Metabolites

**Figure 5:**
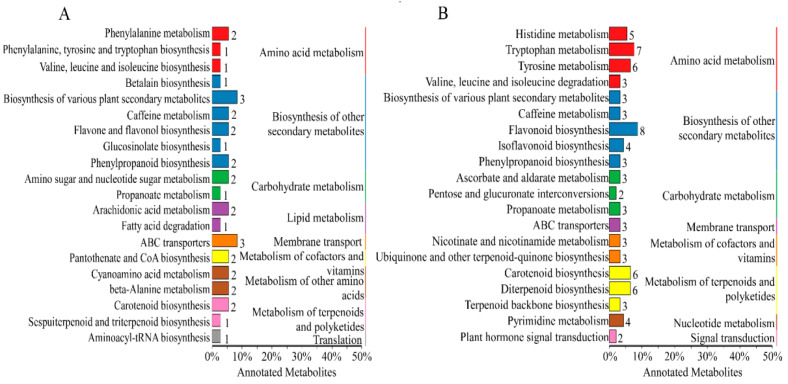
Classification of different metabolite pathways in each group. (**A**) Positive ion model; (**B**) Negative ion model.

Enrichment analysis and overrepresentation analysis (ORA) of the metabolic pathways represented by different metabolites were conducted in the Kyoto Encyclopedia of Genes and Genomes (KEGG) database, and it was found that different metabolites in positive and negative ion modes were enriched in amino acid metabolism, secondary metabolite synthesis, carbohydrate metabolism, terpenoid metabolism pathways, and polyketone metabolism pathways (Figure 5).

The components of bitter taste (bitter substances) include phenolic acids, flavonoids, alkaloids, amino acids, and their polypeptides. The KEGG analysis showed that 55 differential bitter metabolites (of which 50 were upregulated and 5 were downregulated) (Table 2) were enriched under positive and negative ion modes. The enrichment factors of differential metabolites in positive and negative ion models are shown in Figure S4. There were 2, 7, 6, 5, 1, 2, 6, 1, 2, 4, 4, 5, 2, and 8 differential metabolites in phenylalanine metabolism; tryptophan metabolism; tyrosine metabolism; histidine metabolism; phenylalanine, tyrosine and tryptophan biosynthesis; valine, leucine and isoleucine degradation; diterpenoid biosynthesis; sesquiterpenoid and triterpenoid biosynthesis; terpenoid backbone biosynthesis; pyrimidine metabolism; phenylpropanoid biosynthesis; caffeine metabolism; flavone and flavonol biosynthesis; and flavonoid biosynthesis, respectively. The order of their enrichment was defined as follows: amino acid metabolism > flavonoid metabolism > alkaloid metabolism > terpenoid metabolism. The bitter taste of chestnut rose juice caused following OFM feeding may be caused by these differential bitter metabolites.

Bitterness is a sensory attribute that is difficult for the public to accept. Bitterness is usually detected by the stimulation of bitterness receptors (TAS2Rs or T2Rs) in the tongue or mouth, which initiates bitterness signal transduction pathways transmitting bitter taste signals to the brain, and the subsequent perception of bitterness [13,14,15,16]. To avoid ingesting toxic and potentially harmful foods, most organisms have evolved sensitive taste receptors for bitterness. Of course, bitterness is not necessarily toxic, and there are some excellent natural foods with strong bitter tastes, such as coffee, cocoa beans, and bitter melon, which are typical bitter foods, but the proportion of bitter foods that are edible is extremely low. Current studies have shown that bitter substances are widely found in plants, fruits, and vegetables, and play a crucial role in defence against external stress [8]. Kant et al. [17] believed that the defence strategy adopted by plants against pests is to change its nutrient contents and release plant hormones and secondary metabolites. Soluble sugars and amino acids are the main osmotic regulatory substances of plants under stress conditions [18,19]. In plants, pest feeding triggers the defence response of plants, affecting the composition of sugars, amino acids, and other nutrients, thus preventing phytophagous insect feeding [20]. Soluble proteins in plants are generally enzymes involved in metabolism. Plants adapt to unfavourable environments through increasing soluble proteins involved in osmotic regulation [21,22]. The release of plant hormone signalling molecules promotes secondary metabolite transfer, and regulates and induces defence against pests. Jasmonic acid and methyl jasmonate play an important role in balancing plant growth and development and responding to stress. Under pest stress, they regulate the expression of defence genes and produce specific inducer proteins to effectively improve the plant defence response to pests [23,24,25]. Studies have shown that the growth, reproduction, and survival rate of phytophagous insects are related to the release of plant secondary metabolites. Phytophagous insect feeding caused plants to produce toxic secondary metabolites such as phenols by regulating the expression of biosynthetic genes. Excessive intake of phenols by insects results in restricted growth and development and increased larval mortality. In addition, the increased intake of tannins and condensed tannins also impairs insect feeding and digestion, subsequently inhibiting the invasion of pests [26,27]. Under pest stress, changes in plant nutrients participate in a series of physiological and biochemical reactions to enhance their defensive abilities.

The secondary metabolites of plants can affect the feeding, growth, development, and reproduction of pests, attract the natural enemies of pests, and alter the biological characteristics of the population, resulting in direct or indirect defence against pests. There are many kinds of plant secondary metabolites related to pest resistance, such as terpenoids, phenolic compounds, and a variety of nitrogen-containing compounds, including alkaloids and amines. S. Mitra et al. [9] claimed that the activities of phenolic compounds (such as guaiacol and pyrogallol) of *Ludwigia prostrata* Roxb. were significantly increased under *Altica cyanea* feeding stress. Bosch Marko et al. [8] reported that the feeding of beet armyworm (*Spodoptera exigua*) on tomato leaves triggered a defence mechanism, leading to increased levels of monoterpenoids and sesquiterpenoids. Phenols and terpenoids are naturally bitter substances in plants [28,29] that are capable of protecting plants from damage caused by pests. Free amino acids not only play a significant role in plant resistance to stress, but are also important taste substances. Bitterness is conferred by the interaction between hydrophobic side chains of free amino acids and bitterness receptors on the human tongue [30]. In this study, amino acids in chestnut rose juice were identified, and it was found that seven (histidine, leucine, tyrosine, isoleucine, lysine, valine and phenylalanine) out of fifteen amino acids were bitter [31,32]. Although the increased proportion of bitter amino acids, caused by decreased contents of umami and sweet amino acids, enhanced the bitterness of chestnut rose juice, the recognition threshold of these bitter amino acids is relatively high. The DoT coefficient of histidine was more than 1 (DoT = 1.66), which may be one of the reasons for the bitterness of chestnut rose juice, and the other six bitter amino acids all had DoT coefficients less than 1, so the overall bitterness enhancement effect of these amino acids was not significant [31,33]. Combined with the increase in soluble proteins, it was suspected that the chestnut rose may be invaded by OFM, and its defence enzyme system may be activated to promote amino acid synthesis of pest-resistant or bitter proteins, thereby enhancing pest resistance.

Metabolites are the final products in the process of cell signal transmission, and are important contributors to the flavour, functional characteristics, and physical properties of foods. Differential metabolite analysis facilitates understanding the mechanism of biological responses to stress [34,35]. In this study, nontargeted metabolomics were used to analyse the metabolites between normal chestnut rose juice and those that were subjected to OFM feeding stress. Different metabolites were identified based on the classification of bitter substances and the screening parameters used. Then, the metabolic pathways in which each metabolite was enriched were obtained. The results showed that the enrichment of bitter compounds involved 14 metabolic pathways, mainly flavonoid biosynthesis, tryptophan metabolism, tyrosine metabolism, and diterpenoid biosynthesis. Flavonoids have functions such as adjusting auxin transport, regulating reactive oxygen species, and preventing ultraviolet irradiation [36], which not only affects fruit colour and flavour, but also help plants resist biological and abiotic stresses such as low temperatures and pathogenic bacteria [37,38]. In the flavonoid biosynthesis metabolic pathway, there were eight flavonoid metabolites, including xanthohumol, pseudobaptigenin, coumestrol, butin, liquiritigenin, (+)-gallocatechin, dihydromyricetin, and sakuranetin. Usually, it is thought that flavonoid monomers, such as xanthohumol, (+)-gallocatechin, and sakuranetin, are bitter. These flavonoid monomers can exist freely and also in the form of glycosides or acyl derivatives after undergoing a polymerization reaction with other flavonoids, sugars, and nonflavonoids. The type of glycoside chain determines the intensity of bitterness. Xanthohumol, with a bitterness threshold of 10 μmol/L, is a chalcone with isopentenyl [39] and can give wine pleasant bitterness [40]. Xanthohumol is similar to alpha-acid and can be isomerized under boiling conditions to form isoxanthohumol (8C) and demethylxanthohumol (12C), which are less bitter. Xanthohumol and isoxanthohumol can undergo oxidation reactions to produce many oxidation products that have an impact on the bitterness of wine [41]. 4-Coumarin-CoA reacts with chalcone synthase (CHS) and chalcone reductase (CHR) to generate isoliquiritigenin, which is isomerized into liquiritigenin by chalcone isomerase (CHI). Liquiritigenin can reduce bitterness without introducing odour [36]. A potent phytoestrogen, coumestrol, with the skeleton of coumarin and isoflavone, has a good regulatory effect on oestrogen receptors [42]. It is also an effective α-glucosidase inhibitor that can exert antioxidant properties by providing hydrogen atoms or electron transfer. Studies have found that the iron-reducing capacity of coumestrol is approximately twice that of other isoflavones and only half that of quercetin. Rutin (7,3′,4′-trihydroxy-dihydroflavone) has an excellent free radical scavenging ability, and its activity is maintained when combined with serum albumin [43], which contributes to the antioxidant function of chestnut rose.

In plants, tryptophan and tyrosine are produced by erythritosaccharide tetraphosphate and phosphoenolpyruvate under the action of chorismic acid [44], which are not only synthetic components of proteins, but are also situated upstream of secondary metabolites and growth hormones, playing a role in protecting plants from biological and abiotic stresses [45]. Tryptophan (beta-indolyl alanine) is an essential amino acid, and tastes slightly bitter. In the tryptophan metabolism pathway, 5-hydroxyindoleacetate, formyl-5-hydroxykynurenine, L-formylkynurenine, 3-hydroxyanthranilic acid, 5-hydroxykynurenine, 2-aminophenoxazin-3-one, and 3-hydroxykynurenine were enriched. Tryptophan, as a precursor of formyl-5-hydroxycanuridine, L-formylcanuridine, and 3-hydroxycanuridine, plays a key role in the regulation of plant growth and development, their immune response, and oxidative stress. It is also a precursor to the synthesis of glucosinolates, which are naturally bitter plant products derived from amino acids [46]. Tryptophan collaborates with threonine, lysine, and methionine to improve the nutritional quality of plant-derived foods [45]. Tyrosine (2-amino-3-p-hydroxyphenylpropionic acid) is an aromatic polar alpha-amino acid with a phenolic hydroxyl group and tastes bitter. It is the hub of a variety of metabolic pathways, as well as the precursor of special metabolites such as vitamin E, isoquinoline alkaloids, and partial phenylpropanol, which have many physiological effects, such as serving as defence compounds, attractants, and nonprotein amino acids [45,47]. In the tyrosine metabolism pathway, gentisic acid, 5-(L-alanine-3-yl)-2-hydroxy-cis, cis-muconate 6-semialdehyde, gentisate aldehyde, acetoacetate, 4-coumarate, and hydroquinone were enriched. Gentisic acid is an isomer of protocatechuic acid with a good reducing ability [48]. Its biosynthetic precursor, salicylic acid, is a defence-signalling molecule that causes plant defence genes to activate signalling molecules under the stress of infectious pathogens and adverse environments, thus promoting the accumulation of salicylic acid and gentian acid, as well as enhancing plant resistance [49]. 4-Coumarate is also known as p-coumaric acid. In the tyrosine metabolic pathway, tyrosine catalysed by tyrosine ammonia-lyase (TAL) produces p-coumaric acid [50] and forms umbelliferone. Then, a bitter substance, coumarin, is formed with the substitution of isopentenyl. Coumarin is recognized by the bitter receptors BmGr16, BmGr53, and BmGr18 of lepidoptera [51]. P-Coumaric acid can be catalysed by 4-coumaryl-Co-A ligase and stilbene synthase to generate resveratrol [52]. Resveratrol is present in the form of slightly bitter resveratrol glycosides in plants, and its bitterness has a deterrent effect on insect feeding.

Colourless needle-like or powdery substances, such as diterpenoids, produce an intense bitter taste without odour characteristics [53]. Diterpenoids are formed by the condensation of four isopentene pyrophosphoric acids and the universal precursor geranylgeranyl diphosphate (GGPP). Under the catalysis of cytochrome P450, the diterpenoid skeleton is modified to form a variety of complex chemical structures with tetracyclic compounds [54]. The diterpenoid biosynthesis pathway is enriched with gibberellin A36, gibberellin A8, ent-copalyl diphosphate, gibberellin A53, GGPP, and gibberellin A29-catabolite. The plant hormone gibberellin is a tetracyclic diterpenoid compound, and its synthetic precursor is ent-kaurene derived from the cyclization of GGPP. Ent-kaurene is oxidized to produce GA12-aldehyde, an important intermediate of gibberellin biosynthesis, and gibberellin A53 is oxidized in the cytoplasmic matrix to form a variety of gibberellins [55]. Free gibberellin is a mono-, di-, or tricarboxylic acid with 19C or 20C, while most bound gibberellin is glucoside or glucosylester. Under the stress of diseases and pests, gibberellin induces resistance responses to enhance the ability of plants to resist stress [56]. In addition, increased gibberellin levels promote the membrane repair of damaged cells to protect plants [57].

## 3. Materials and Methods

### 3.1. Main Reagents

Rutin (7,3′,4′-trihydroxy-dihydroflavone) was purchased from Kuer Bioengineering Co., Ltd., Hefei, China. Sodium molybdate, sodium tungstate, lithium sulfate, and sodium nitrite were obtained from Shanghai Macklin Biochemical Technology Co., Ltd., Shanghai, China. Gallic acid monohydrate was purchased from Tianjin Kemio Chemical Reagent Co., Ltd., Tianjin, China. 2-Chloro-L-phenylalanine (≥98%) was purchased from Shanghai Aladdi Co., Ltd., Shanghai, China.

### 3.2. Collection and Juicing of Chestnut Rose Fruit

Fresh chestnut rose fruit, “Gui Nong No. 5”, was collected from the National Chestnut Rose Demonstration Park in Longli County, Qiannan Buyi, and Miao Autonomous Prefecture, Guizhou Province, China (altitude of 900~1500 m, organic matter content ≥ 2.0%, pH 5.0~7.0). The park was divided into five regions: east, west, south, north, and middle. Five samples were selected from each region using the diagonal method, each sample was ≥20 kg, and a total of 25 samples were sampled from 5 regions. Before juicing, each fruit was divided into two parts with a knife (at 4 °C temperatures) to observe the “blackened” phenomenon inside them. If it is “blackened”, it is identified as a worm fruit (eaten by OFM) (Figure S1B); If it is not “blackened”, it is identified as a normal fruit (Figure S1A). The processing of chestnut rose juice adopts a low-temperature physical pressing method (in a press machine), pre-treatment does not crush without adding pectinase, cellase, and other enzymes, and the juice is filtered and centrifuged (at 4 °C and 12,000 rpm for 15 min) after squeezing. Then, the supernatant was packed in a high-barrier aluminium foil BIB bag and stored at −20 ℃ (normal juice was marketed as “G”; the juice extracted from the chestnut rose fruits eaten by OFM was marked as “B”, n = 25). To ensure that the data in the experiment are representative, we mixed 10 samples from the east and west regions, 10 samples from the north and south regions, and 5 samples from the middle region into 1 sample according to the principle of the diagonal two tops, so as to make 3 sample replicates.

### 3.3. Determination of the Functional Active Ingredients in Chestnut Rose Juice

Chestnut rose was weighed by an electronic balance (SB 3003, Haining Shengbo Weighing Instrument Co., Ltd., Ningbo, China) The transverse and longitudinal diameters were measured by a Vernier calliper (0–150 mm, Yantai Lvlin Tool Co., Ltd., Yantai, China). The total colour difference was determined using a high-quality computer colour difference metre (SC-10, Shenzhen Sanenshi Technology Co., Ltd., Shenzhen, China). The soluble solid content was determined using a handheld refractometer (WYT-Ⅱ, Optical Instrument Department of Chengdu Youth Federation). Superoxide dismutase (SOD) levels were determined according to the instructions of the SOD assay kit (Nanjing Jiancheng Bioengineering Institute). Total protein was determined with a BCA protein assay kit (Beyotime Biotechnology, Shanghai, China). The vitamin C content was determined using molybdenum blue colorimetry [58]. The tannin content was determined using spectrophotometry in accordance with NY/T1600-2008. Condensed tannins were detected using a vanillin reaction [59]. Polyphenols were determined using the Folin-phenol method [60]. Flavonoid content was determined using the aluminium nitrate chromogenic method [61].

### 3.4. Electronic Tongue Test

The multifunctional taste nutrition detection system was implemented using the electronic tongue SA402B of Insent company in Japan (TS-5000Z, Insent Company, Fukuoka, Japan). This system has eight taste sensors: sour taste, bitter taste, astringent taste, aftertaste-B (bitter aftertaste), aftertaste-A (astringent aftertaste), umami taste, umami aftertaste, and salty taste. Chestnut rose was mechanically pressed for juice, and the juice was filtered and centrifuged at 4 °C and 12,000 rpm for 15 min. Then, the supernatant was absorbed for electronic tongue testing. Before the detection began, the taste sensor and reference electrode were pretreated to stabilize the potential; all sensors, reference electrodes (3.33 mol/L KCl solution), and the internal solution (3.33 mol/L KCl + saturated silver chloride) were connected to the sensor connector. The reference solution was composed of a 30 mmol/L KCl solution and 0.3 mmol/L of tartaric acid. The negatively charged membrane washing solution was applied to the negatively charged membrane sensors AC0, AN0, and BT0, as well as the hybrid membrane sensors AAE, CT0, and CA0. The positively charged membrane washing solution was applied to the positively charged membrane sensors C00, AE1, and GL1.

### 3.5. Amino Acid Profile Analysis

The automatic amino acid analyser L-8800 (Hitachi, Tokyo, Japan) was used to investigate the amino acid spectrum of chestnut rose juice. Sample collection and preparation were the same as in the electronic tongue test. Preparation of the amino acid standard curve: 0.2 mL of the mixed amino acid standard was diluted with a buffer (pH 2.2) to a concentration of 5.0 nmol/50 μL. The automatic amino acid analyser went as follows: chromatographic column (4.6 mm ID × 60 mm), packing material (3 μm ion exchange resin), injection volume (0.05 mL), velocity of channel 1 (0.4 mL/min), velocity of channel 2 (0.35 mL/min); the analysis time of each sample was set to 60 min.

### 3.6. Nontargeted Metabolomics Analysis

An appropriate amount of chestnut rose juice was placed into the extraction solution (equal volume of methanol and acetonitrile, internal standard concentration: 2 mg/L, subjected to ultrasound for 10 min in ice-water bath) and incubated for 1 h at −20 °C. Then, the samples were centrifuged at 4 °C, and 12,000 rpm for 15 min. Then, 500 μL of supernatant was absorbed into an Eppendorf tube and subjected to vacuum drying. An extract solution (160 μL; equal volume of acetonitrile and water) was added and the mixture was vortexed for 30 s, subjected to ultrasound for 10 min in an ice-water bath, and centrifuged at 4 °C and 12,000 rpm for 15 min. Then, 120 μL of supernatant was placed into 2 mL sample bottles, and 10 μL of each sample was mixed into the QC samples for LC-QTOF detection.

The liquid mass system for untargeted metabolomics analysis comprised ultrahigh-performance liquid chromatography (Waters UPLC Acquity I-Class PLUS, Waters, Milford, MA, USA) and a high-resolution mass spectrometer (Waters UPLC Xevo G2-XS QTOF, Waters, Milford, MA, USA). The chromatographic column (Acquity UPLC HSS T3: 2.1 × 100 mm × 1.8 μm, Waters, Milford, MA, USA) [62] was loaded with 1 μL of the sample volume. The electron spray ionization (ESI) source configured by the mass spectrometer had both positive ion (+) (POS) and negative ion (−) (NEG) modes. The POS included mobile phase A (0.1% formic acid aqueous solution) and mobile phase B (0.1% formic acid acetonitrile). The NEG included mobile phase A (0.1% formic acid aqueous solution) and mobile phase B (0.1% formic acid acetonitrile). The conditions of the mobile phase in positive and negative ion modes were the same: the flow rate was always 400 μL/min; at 0 min and 0.25 min, 98% A solution and 2% B solution; 10 min and 13 min, 2% A solution and 98% B solution; 13.1 min and 15 min, 98% A solution and 2% B solution.

Primary and secondary mass spectrometry data were collected using the MSe mode controlled by the acquisition software of the high-resolution mass spectrometer (MassLynx V4.2, Waters, Milford, MA, USA). In each data acquisition cycle, dual-channel data acquisition was conducted for both low impact energy (2 V) and high impact energy intervals (10~40 V), with a scanning frequency of 0.2 s per mass spectrum. The ESI source parameters were as follows [62]: cone-hole voltage (30 V), ion source temperature (100 °C), desolvation temperature (500 °C), blowback gas flow (50 L/h), desolvation gas flow (800 L/h), and collection range of mass-to-charge ratio (50–1200 *m*/*z*). The capillary voltages of the POS and NEG were 2.5 kV and 2.0 kV, respectively.

### 3.7. Data Processing and Statistics

In nontargeted metabolomics analysis, original data collected by MassLynx V4.2 software were processed by Progenesis QI 2.1 software for peak extraction and peak alignment, and the metabolites were identified using Progenesis QI software, online BMKCloud, and METLIN databases. Flavour biosynthetic pathways were explored through Kyoto Encyclopedia of Genes and Genomes (KEGG) analysis, and theoretical fragment recognition was performed to normalize the total peak area of the data. The mass number deviation of the parent ion was within 100 ppm, and that of the fragment ion was within 50 ppm [62]. This means that the ratio of each metabolite in the total peak area of the sample is multiplied by the mean of all peak areas.

Univariate statistical analysis (*t*-test) and multivariate statistical analysis (including principal component analysis, PCA; fold change, FC; and orthogonal partial least square discriminant analysis, OPLS-DA) were performed through a metabolomic information analysis process using R-3.3.1 software for metabolite classification and functional annotation. PCA was used to investigate the variation between different groups and between samples within groups. After log2 conversion of data, an OPLS-DA model was constructed between comparative analysis groups, with 7-fold cross-validation. To ensure the reliability of the OPLS-DA model, a replacement test (200 times) was needed. The VIP (variable importance projection) values of metabolites were obtained by OPLS-DA, thus initially screening out differential metabolites. The differential metabolites were further filtered by the p value or fold change value (FC) of univariate analysis. The screening criteria were set to *p* value < 0.05, VIP value ≥ 1, and FC ≥ 1 or <0.5. The upregulation and downregulation of differential metabolites were determined based on the G-mean value.

## 4. Conclusions

This study analysed the changes in flavour substances in chestnut rose juice produced by fruits subjected to OFM feeding stress. The results indicated that OFM feeding altered the levels of nutrients and amino acids in chestnut rose fruit, and the release of secondary metabolites and plant hormones enhanced the bitterness of chestnut rose fruit to resist OFM feeding. Moreover, this study revealed for the first time the material basis for and metabolic pathway of flavour deterioration in chestnut rose fruit subjected to OFM feeding stress. These results provide a theoretical basis for the study of quality deterioration in other fruit products under pest stress and support the value of conducting further research on the planting, management, and processing of high-quality chestnut rose.

## Figures and Tables

**Table 1:** Basic indexes of chestnut rose fruit and bioactive components of its juice.

Index	G	B
Weight (g)	20.34 ± 4.37 ^a^	21.88 ± 4.05 ^a^
Transverse and longitudinal ratio	1.37 ± 0.13 ^a^	1.30 ± 0.12 ^b^
Total color difference	52.40 ± 2.20 ^a^	52.55 ± 1.77 ^a^
Juice yield (%)	67.19 ± 1.05 ^a^	55.36 ± 5.95 ^b^
Bad fruit rate (%)	27.68 ± 1.27	
Soluble solid (%)	9.39 ± 0.06 ^b^	11.07 ± 0.06 ^a^
SOD (U/mL)	118.41 ± 1.04 ^a^	117.23 ± 0.51 ^a^
Protein concentration (mg/mL)	20.64 ± 0.52 ^b^	22.81 ± 1.24 ^a^
Vitamin C (mg/100 g)	2038.93 ± 134.36 ^a^	1971.22 ± 139.85 ^a^
Tannin (mg/mL)	4.47 ± 0.12 ^a^	4.12 ± 0.21 ^a^
Consented tannin (mg/mL)	36.62 ± 0.87 ^a^	28.35 ± 5.64 ^a^
Polyphenol (mg/mL)	1.91 ± 0.12 ^a^	1.93 ± 0.02 ^a^
Flavone (mg/mL)	25.15 ± 0.97 ^a^	17.93 ± 0.68 ^b^

Note: Significant level *p* < 0.05. When there is significance between groups, the large number is marked with “a” and the small number is marked with “b”.

**Table 2:** Potential bitter metabolites screened out by untargeted metabolomics.

Pathway	Metabolite	Formula	*p*	VIP	Fold Change	Regulated
Phenylalanine metabolism	Phenylacetyl-CoA	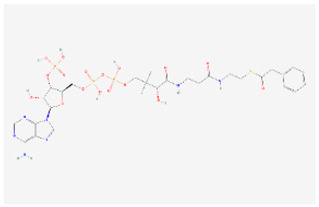 C_29_H_42_N_7_O_17_P_3_S	0.003	1.039	1.572	Up
	2-Hydroxyphenylacetate	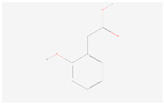 C_8_H_8_O_3_	0.005	1.025	1.462	Up
Tryptophan metabolism	5-Hydroxyindoleacetate	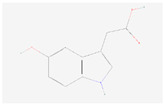 C_10_H_9_NO_3_	0.004	1.019	1.626	Up
	Formyl-5-hydroxykynurenamine	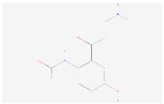 C_10_H_12_N_2_O_3_	0.006	1.020	1.245	Up
	L-Formylkynurenine	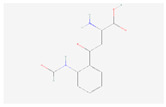 C_11_H_12_N_2_O_4_	0.001	1.024	1.199	Up
	3-Hydroxyanthranilic acid	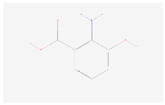 C_7_H_7_NO_3_	0.012	1.021	1.227	Up
	5-Hydroxykynurenamine	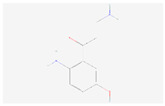 C_9_H_12_N_2_O_2_	0.000002	1.052	0.460	Down
	2-Aminophenoxazin-3-one	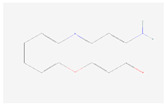 C_12_H_8_N_2_O_2_	0.012	1.002	1.392	Up
	3-Hydroxykynurenamine	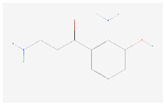 C_9_H_12_N_2_O_2_	0.0005	1.045	2.288	Up
Tyrosine metabolism	Gentisic acid	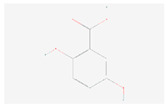 C_7_H_6_O_4_	0.002	1.019	1.169	Up
	5-(L-Alanin-3-yl)-2-hydroxy-cis,cis-muconate 6-semialdehyde	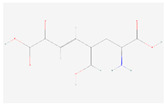 C_9_H_11_NO_6_	0.001	1.042	1.118	Up
	Gentisate aldehyde	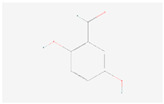 C_7_H_6_O_3_	0.016	1.014	1.084	Up
	Acetoacetate	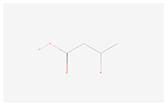 C_4_H_6_O_3_	0.001	1.026	1.446	Up
	Hydroquinone	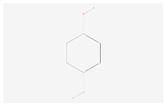 C_6_H_6_O_2_	0.007	1.011	1.449	Up
	4-Coumarate	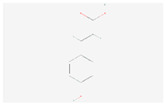 C_9_H_8_O_3_	0.000004	1.052	1.780	Up
Histidine metabolism	N-Formimino-L-glutamate	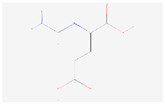 C_6_H_10_N_2_O_4_	0.002	1.031	1.352	Up
	L-Histidinol phosphate	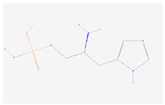 C_6_H_12_N_3_O_4_P	0.011	1.006	1.820	Up
	Dihydrourocanate	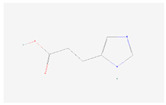 C_6_H_8_N_2_O_2_	0.005	1.017	1.163	Up
	Hydantoin-5-propionate	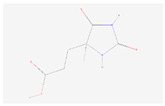 C_6_H_8_N_2_O_4_	0.0003	1.038	1.357	Up
	D-erythro-1-(Imidazol-4-yl)glycerol 3-phosphate	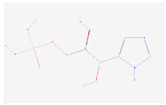 C_6_H_11_N_2_O_6_P	0.001	1.046	3.296	Up
Phenylalanine, tyrosine, and tryptophan biosynthesis	Quinate	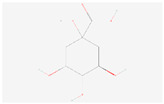 C_7_H_12_O_6_	0.013	1.023	0.440	Down
Valine, leucine, and isoleucine degradation	2-Methyl-1-hydroxypropyl-ThPP	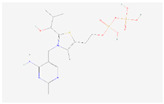 C_16_H_27_N_4_O_8_P_2_S^+^	0.006	1.033	1.450	Up
	(S)-3-Methyl-2-oxopentanoic acid	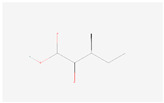 C_6_H_10_O_3_	0.006	1.036	1.225	Up
Diterpenoid biosynthesis	Gibberellin A36	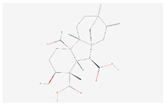 C_20_H_26_O_6_	0.0004	1.048	1.199	Up
	Gibberellin A8	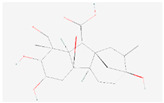 C_19_H_24_O_7_	0.001	1.025	1.404	Up
	ent-Copalyl diphosphate	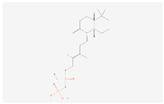 C_20_H_36_O_7_P_2_	0.002	1.035	1.265	Up
	Gibberellin A53	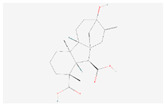 C_20_H_28_O_5_	0.00006	1.051	1.643	Up
	Gibberellin A29-catabolite	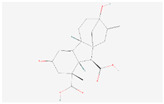 C_19_H_22_O_6_	0.017	1.012	1.433	Up
	Geranylgeranyl diphosphate	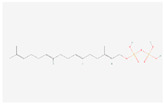 C_20_H_36_O_7_P_2_	0.005	1.012	1.242	Up
Sesquiterpenoid and triterpenoid biosynthesis	(−)-Germacrene D	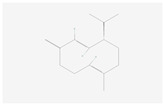 C_15_H_24_	0.001	1.050	2.007	Up
Terpenoid backbone biosynthesis	all-trans-Hexaprenyl diphosphate	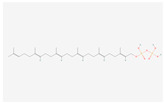 C_30_H_52_O_7_P_2_	0.002	1.016	2.209	Up
	4-(Cytidine 5′-diphospho)-2-C-methyl-D-erythritol	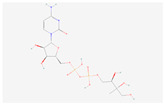 C_14_H_25_N_3_O_14_P_2_	0.006	1.010	0.377	Down
Pyrimidine metabolism	dCMP	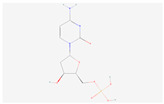 C_9_H_14_N_3_O_7_P	0.013	1.019	1.184	Up
	Pseudouridine	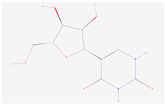 C_9_H_12_N_2_O_6_	0.013	1.008	1.166	Up
	3-Hydroxypropanoate	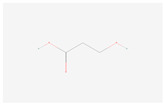 C_3_H_6_O_3_	0.0003	1.043	1.312	Up
	Cytidine	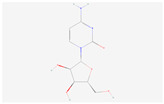 C_9_H_13_N_3_O_5_	0.009	1.015	1.175	Up
Phenylpropanoid biosynthesis	Caffeyl alcohol	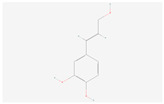 C_9_H_10_O_3_	0.014	1.019	1.462	Up
	1-O-Sinapoyl-beta-D-glucose	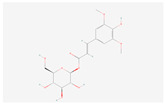 C_17_H_22_O_10_	0.001	1.045	1.331	Up
	5-Hydroxyconiferaldehyde	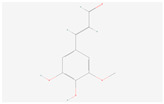 C_10_H_10_O_4_	0.004	1.066	1.252	Up
	Coniferyl aldehyde	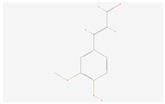 C_10_H_10_O_3_	0.003	1.026	1.249	Up
Caffeine metabolism	7-Methylxanthine	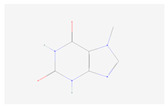 C_6_H_6_N_4_O_2_	0.007	1.008	1.548	Up
	N,N’-Dimethylurea	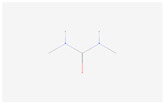 C_3_H_8_N_2_O	0.006	1.029	1.487	Up
	1-Methyluric acid	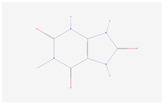 C_6_H_6_N_4_O_3_	0.001	1.031	1.530	Up
	5-Acetylamino-6-formylamino-3-methyluracil	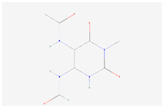 C_8_H_10_N_4_O_4_	0.004	1.043	2.943	Up
	7-Methylxanthosine	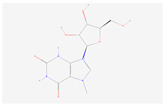 C_11_H_15_N_4_O_6_	0.0004	1.038	3.267	Up
Flavone and flavonol biosynthesis	Quercetin 3-sulfate	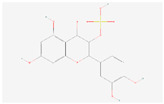 C_15_H_10_O_10_S	0.004	1.066	0.466	Down
	Lampranthin II	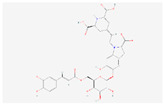 C_34_H_34_N_2_O_16_	0.003	1.049	0.426	Down
Flavonoid biosynthesis	Xanthohumol	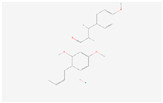 C_21_H_22_O_5_	0.0005	1.044	1.336	Up
	Pseudobaptigenin	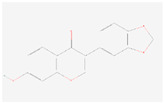 C_16_H_10_O_5_	0.007	1.031	1.512	Up
	Coumestrol	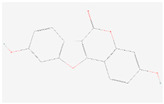 C_15_H_8_O_5_	0.00007	1.053	1.193	Up
	Butin	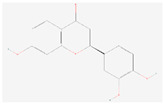 C_15_H_12_O_5_	0.004	1.004	1.143	Up
	Liquiritigenin	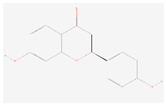 C_15_H_12_O_4_	0.006	1.004	1.282	Up
	(+)-Gallocatechin	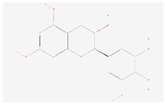 C_15_H_14_O_7_	0.004	1.002	1.195	Up
	Dihydromyricetin	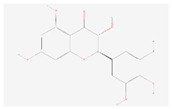 C_15_H_12_O_8_	0.003	1.023	1.969	Up
	Sakuranetin	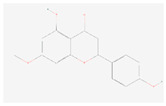 C_16_H_14_O_5_	0.00007	1.046	2.763	Up

Note: The difference-accumulating compounds were identified by *t*-test, *p* < 0.05 (significant), FC ≥ 1 (up-regulated), FC < 0.5 (down-regulated), VIP ≥ 1.

## Data Availability

The data are available from the corresponding author on reasonable request.

## Supplementary Materials

The following supporting information can be downloaded at: https://www.mdpi.com/article/10.3390/molecules28207170/s1, Figure S1: Appearance of chestnut rose. (A) the normal chestnut rose fruit; (B) the worm chestnut rose fruit; Figure S2: PCA analysis of metabolites identified from ‘G’ and ‘B’.(A) Positive ion model; (B) Negative ion model; Figure S3: Permutation test of OPLS-DA.(A) Positive ion model; (B) Negative ion model; Figure S4: Enrichment factors of differential metabolites in positive and negative ion models.(The X-axis is the enrichment factor (Rich factor) of differential metabolites enriched in the pathway; The Y-axis is the P-value pathway; Dot size represents the number of differentially expressed metabolites annotated to this Pathway). (A) Positive ion model; (B) Negative ion model.

## Sample Availability

Samples of the compounds are not available.
