# Supplementary material for: Research on the Effect of Oriental Fruit Moth Feeding on the Quality Degradation of Chestnut Rose Juice Based on Metabolomics
Source: Molecules. 2023 Oct 19;28(20):7170. doi: 10.3390/molecules28207170 (PMC10608842; doi:10.3390/molecules28207170)
Supplement: Supplementary file 1 [file molecules-28-07170-s001.zip › molecules-2592989-supplementary.pdf]

FIG. S1:

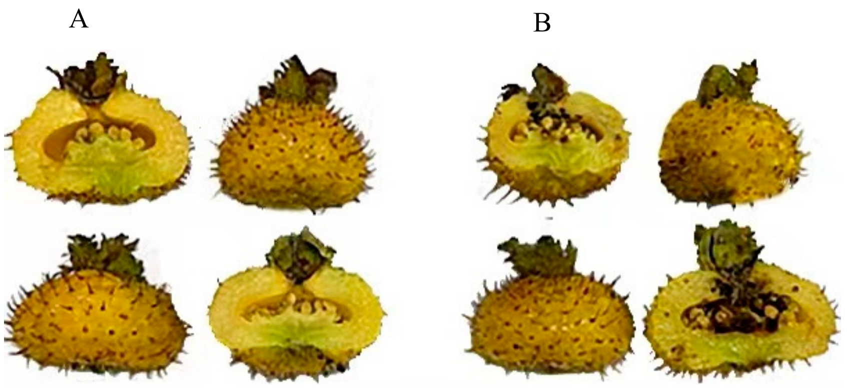

FIG. S1 Appearance of chestnut rose. (A) the normal chestnut rose fruit; (B) the worm chestnut rose fruit.

FIG. S2:

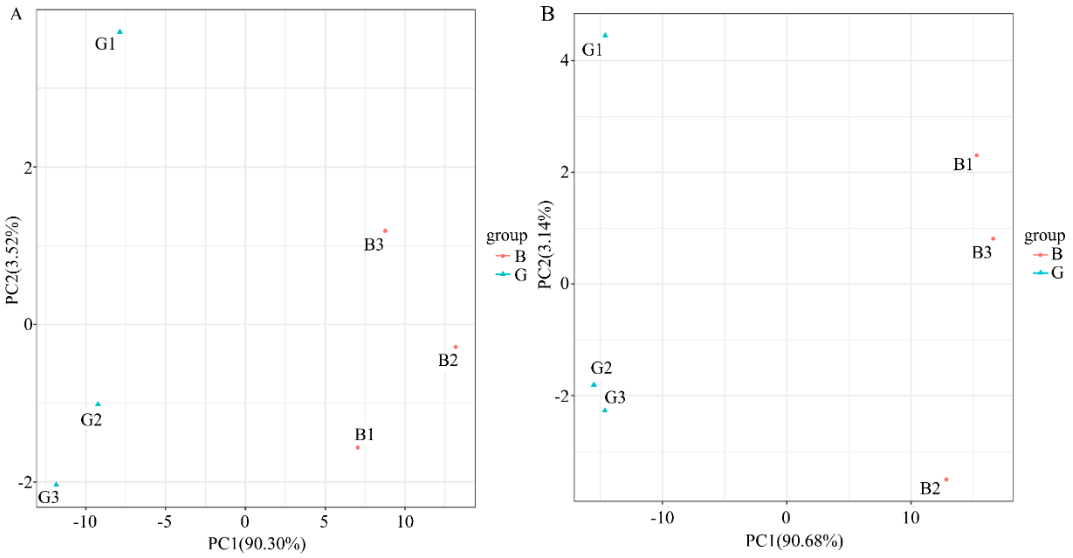

FIG. S2 PCA analysis of metabolites identified from 'G' and 'B'.(A) Positive ion model; (B) Negative ion model.

FIG. S3:

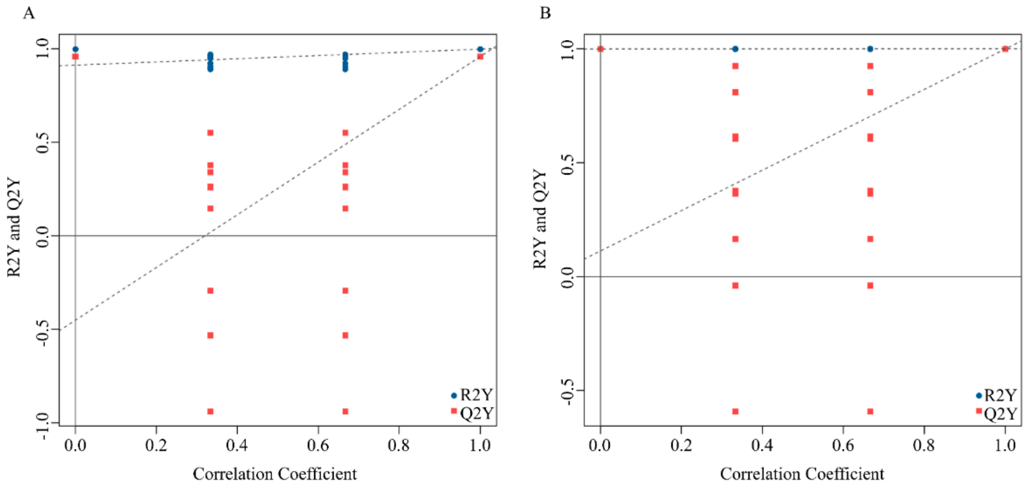

FIG. S3 Permutation test of OPLS-DA.(A) Positive ion model; (B) Negative ion model.

FIG. S4:

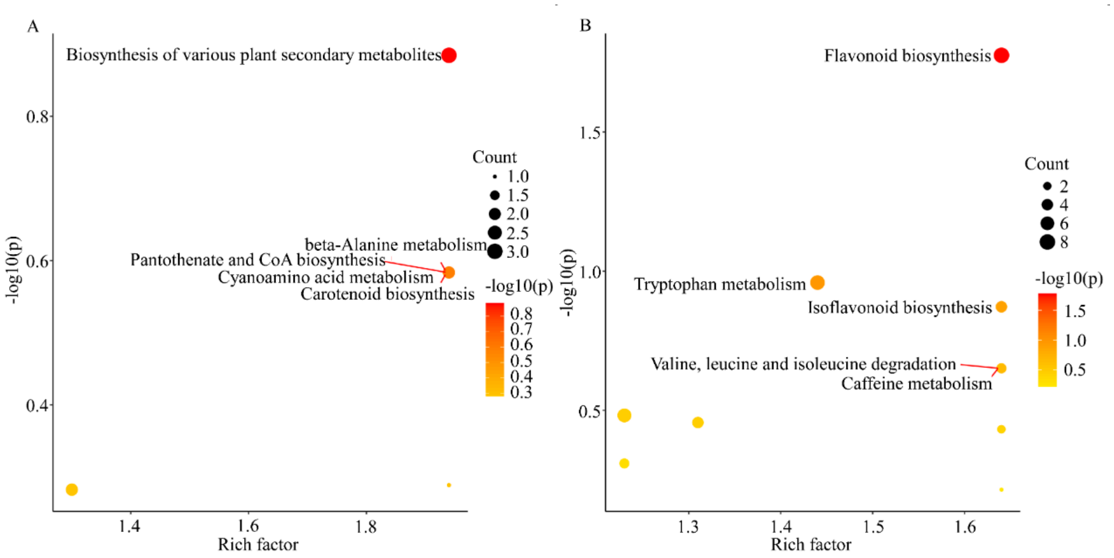

FIG. S4. Enrichment factors of differential metabolites in positive and negative ion models.（The X-axis is the enrichment factor (Rich factor) of differential metabolites enriched in the pathway; The Y-axis is the P-value pathway; Dot size represents the number of differentially expressed metabolites annotated to this Pathway. (A) Positive ion model; (B) Negative ion model.
